# Effects of *Sous-Vide* on Quality, Structure and Flavor Characteristics of Tilapia Fillets

**DOI:** 10.3390/molecules28248075

**Published:** 2023-12-13

**Authors:** Luqian Yang, Zhaoyong Li, Tianxiang Xie, Jun Feng, Xinxing Xu, Yuanhui Zhao, Xin Gao

**Affiliations:** 1 Sanya Oceanographic Institution, Ocean University of China, Sanya 572025, China; yyyluqian@sina.com (L.Y.); xtx920029414@126.com (T.X.); 15734990829@163.com (J.F.); zhaoyuanhui@ouc.edu.cn (Y.Z.); 2 Qingdao Institute of Measurement Technology, Qingdao 266000, China; 3 College of Food Science and Engineering, Ocean University of China, Qingdao 266404, China; xxx@ouc.edu.cn

**Keywords:** tilapia, *sous-vide* cooking technique, quality change, flavor

## Abstract

To investigate the effects of traditional high-temperature cooking and *sous-vide* cooking on the quality of tilapia fillets, muscle microstructure, texture, lipid oxidation, protein structure, and volatile compounds were analyzed. In comparison with samples subjected to traditional high-temperature cooking, *sous-vide*-treated samples exhibited less protein denaturation, a secondary structure dominated by α-helices, a stable and compact structure, a significantly higher moisture content, and fewer gaps in muscle fibers. The hardness of the *sous-vide*-treated samples was higher than that of control samples, and the extent of lipid oxidation was significantly reduced. The *sous-vide* cooking technique resulted in notable changes in the composition and relative content of volatile compounds, notably leading to an increase in the presence of 1-octen-3-ol, α-pinene, and dimethyl sulfide, and a decrease in the levels of hexanal, D-limonene, and methanethiol. *Sous-vide* treatment significantly enhanced the structural stability, hardness, and springiness of muscle fibers in tilapia fillets and reduced nutrient loss, enriched flavor, and mitigated effects on taste and fishy odor.

## 1. Introduction

Tilapia is a versatile fish that grows quickly, reproduces easily, adapts well to different environments, and readily consumes artificial feeds, which makes it an ideal choice for aquaculture. China is the leading producer of tilapia and accounts for almost half of the global production. The tilapia production in China experienced a rise from 166,2637 tons in 2021 to 173,8947 tons in 2022, which represented over 45% of the total world production [1]. Therefore, it is crucial for China to improve the quality of tilapia and the economic efficiency of tilapia production. Tilapia fillets are popular among consumers because they are boneless, affordable, and rich in unsaturated fatty acids and protein [2,3]. However, tilapia meat has a highwater content, which makes it prone to spoilage [4]. Freezing is commonly used to extend its shelf life, but it results in a low-value product. Chen et al. [5] investigated the effects of different types of heat treatment on the quality of tilapia muscle. They found that baking resulted in a significant loss of moisture and rapid impairment of flavor in tilapia. Traditional high-temperature steaming led to a softer texture (a decrease in hardness from 1468.49 ± 180.77 g to 479.32 ± 80.28 g) and a reduction in chewability. Microwave treatment caused muscle cells to contract and aggregate, which resulted in severe structural damage. Li et al. [6] demonstrated that air frying caused the oxidation of tilapia proteins and lipids. These studies indicate that traditional high-temperature heating can cause thermal damage to tilapia meat and thus alter its internal structure and cause loss of water, which greatly affects its nutrient content, texture, and flavor. Therefore, it is necessary to develop a gentler processing method for tilapia fillets in order to enhance their eating quality and nutritional value.

The *sous-vide* technique involves placing vacuum-packed products in a cooking vessel with precise temperature control and then cooking (pasteurizing) them according to the desired time and temperature parameters [7]. This method is commonly used for fish, as it maintains the freshness and appearance of seafood while extending its shelf life [8]. Vacuum cooking helps to retain moisture, flavor, and nutrients while preventing oxidation and the generation of off-flavors, and the products have higher organoleptic and textural acceptability [7]. The temperature during cooking is crucial for both safety and quality. In a recent study conducted by Wei et al. [9], it was discovered that sturgeon fillets treated with boiling water at 60 °C for 15 min under vacuum conditions exhibited the most intact structure when observed via optical microscopy. In addition, the study revealed that the cooking loss of sturgeon treated at 100 °C was significantly higher (33.12% ± 0.46%) than that of *sous-vide*-treated samples. This higher cooking loss resulted in the loss of protein and water, which ultimately compromised the juiciness of the product. In a separate investigation conducted by Rupali et al. [10], textural changes in whiteleg shrimp (*Litopenaeus vannamei*) under various *sous-vide* treatment conditions were analyzed. The study determined that the optimal *sous-vide* cooking conditions for whiteleg shrimp comprised a temperature of 81.87 °C and a duration of 13.48 min. Under these conditions, the shrimp exhibited the lowest hardness (7235 g). These results highlight the significance of selecting the appropriate *sous-vide* cooking parameters for achieving the desired texture of whiteleg shrimp. In comparison with traditional high-temperature steaming, *sous-vide* steaming offers several advantages in preserving the quality of food. By precisely controlling the steaming temperature and time, it helps to maintain the cellular structure of the food, minimize protein–protein interactions and gelation, and increase the water holding capacity [11]. In addition, *sous-vide* steaming avoids damage to aroma compounds caused by excessive heating and improves the texture of meat [12]. Previous research has demonstrated the effectiveness of *sous-vide* cooking in preventing damage to aroma compounds, reducing denaturation of heat-sensitive nutrients, and minimizing thermal damage to proteins and lipids caused by high temperatures. Furthermore, it enhances the palatability of meat products [12]. A study showed that the *sous-vide* technique improved the texture and flavor profile of cooked tilapia to some extent under optimal conditions [13]. However, there is a lack of detailed information on the effects of the *sous-vide* technique on the physicochemical properties, flavor, and changes in the nutritional aspects of tilapia fillets. Therefore, there is a clear need for more comprehensive data on the effects of *sous-vide* treatment on changes in the quality of tilapia fillets and the underlying mechanisms.

This study aimed to compare the quality, texture, and flavor of tilapia cooked using the *sous-vide* technique with those after traditional high-temperature cooking. Tilapia meat was used as the raw material for both cooking methods. To begin with, the study measured the physical properties of tilapia fillets cooked using both methods. Next, it examined the extent of lipid oxidation and changes in protein structure in the fillets cooked using the different methods. In addition, the study analyzed the effects of the different cooking methods on the aroma characteristics of the fillets using an electronic nose, headspace solid-phase microextraction–gas chromatography–mass spectrometry (HS–SPME–GC–MS), and headspace–gas chromatography–ion mobility spectrometry (HS–GC–IMS) in combination with sensory evaluation. Overall, the focus of this research was to understand the changes in the nutritional quality and flavor of tilapia fillets during cooking under different conditions, with the aim of utilizing tilapia fillets to achieve the best eating experience. The findings of this study will offer researchers and consumers more choices in terms of tilapia preparation.

## 2. Results and Discussion

### 2.1. Morphological Changes in Tilapia Fillets Subjected to Different Cooking Methods

**Figure 1:**
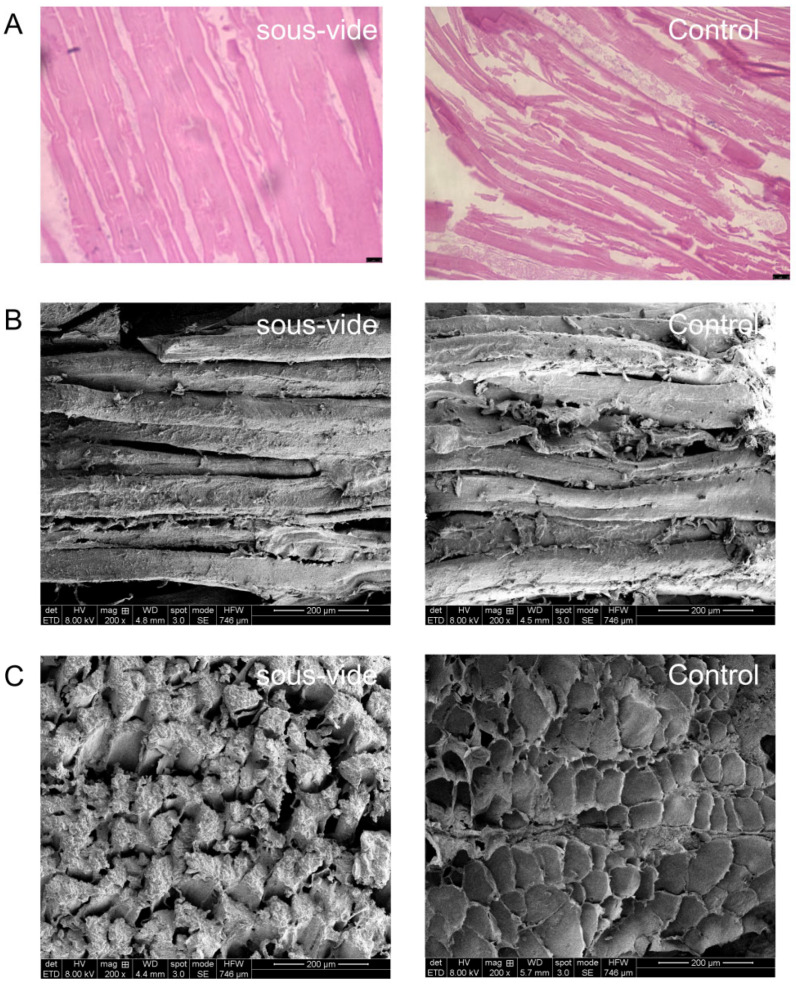
HE staining (**A**), SEM micrographs (**B**) longitudinal section; (**C**) transverse section.

Fillet quality is strongly influenced by muscle microstructure, and the use of H&E staining and scanning electron microscopy for analysis showed that the *sous-vide* process (left) versus traditional cooking at high temperatures (right) was critical to the microstructure of tilapia fillets (Figure 1A–C).

**Figure 2:**
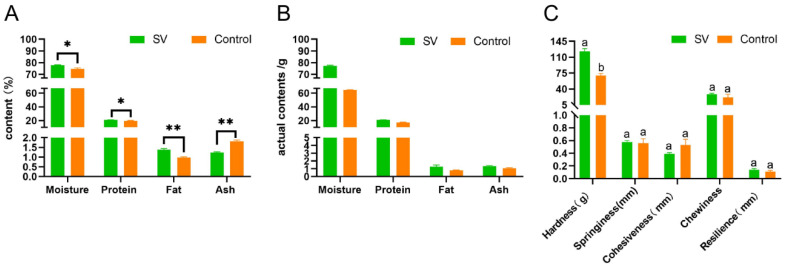
Changes in nutrient composition (**A**,**B**), and texture profile analysis (**C**) of tilapia fillets under different cooking methods. A difference between the letters indicates a significant difference (*p* < 0.05). Levels of significance are shown as follows: * 0.01 < *p* ≤ 0.05; ** 0.001 < *p* ≤ 0.01.

H&E staining showed that the muscle fibers of tilapia after *sous-vide* treatment were in the form of strips arranged longitudinally along the body of the fish and had clear outlines and firm and complete structures, with no obvious traces of breakage or looseness. After cooking at high temperatures, the number of voids in the myofibrils increased and the breakage of some myofibrils worsened and even led to disintegration into fragments. Proteins release water during denaturation, and the effect of moisture may help muscle fibers to align closely, which results in a more compact structure [14]. In comparison, cooking at low temperatures retained more moisture (Figure 2A), and this moisture gave the fillets a compact structure and tightly bound muscle fibers. A more detailed view of the myofibrils and intramuscular connective tissue of tilapia fillets was obtained from scanning electron micrographs. The *sous-vide* samples exhibited a more compact arrangement of fibers, as evidenced by fewer gaps in the myofibrils and a well-organized structure, whereas the samples subjected to conventional high-temperature cooking had a looser structure with extensive extracellular spaces between muscle bundles and larger pores. This structural damage may have contributed to the loss of tenderness and juicy flavor in the fish, which was consistent with the results of sensory evaluation (Figure 1C). During the same period, high temperatures led to the destruction of connective tissue around muscle fibers, which were retracted and lysed and lost their connections and alignment [15]. The *sous-vide* treated fillets exhibited a lower degree of protein denaturation and higher contents of collagen and myofibrillar protein than the group treated by conventional high-temperature cooking. *Sous-vide* treatment was thus more favorable for the tight alignment of muscle fibers and resulted in a more compact structure with less nutrient loss and more water retention. This was consistent with the results of the proximate analyses (Figure 2A,B), which showed that *sous-vide* treated tilapia fillets had higher contents of water and protein than tilapia fillets after high-temperature cooking. In summary, the *sous-vide* process was able to mitigate protein denaturation to some extent and resulted in a denser structure that remained generally ideal, which was consistent with the results of the bulk structure and FTIR spectroscopy measurements. The microstructural changes in sturgeon fillets at different heating temperatures [16] were consistent with the results obtained in this study.

### 2.2. Proximate Analysis of Tilapia Fillets Subjected to Different Cooking Methods

The basic nutrient content is an important indicator of the eating quality of fish but changes with differences in cooking conditions because of processing losses. The basic nutrient contents of tilapia fillets subjected to the different steaming methods are shown in Figure 2A. In comparison with traditional steaming at high temperatures, more proteins and fats were retained in the *sous-vide*-treated fish. The results show that the moisture, crude protein, and crude fat contents of the samples in the *sous-vide* steaming group were significantly higher than those of the samples in the traditional high-temperature steaming group (*p* < 0.05), while the ash content was also higher. The notable reduction in moisture content can primarily be attributed to the evaporation of water at elevated temperatures. In addition, the rise in temperature caused the denaturation and contraction of myofibrillar proteins, which resulted in a diminished capacity to retain water [17]. Consequently, this led to substantial alterations in the protein, fat, and ash composition of the fillets. To account for variations in moisture content among the samples, the weight of the *sous-vide*-treated tilapia fillets was standardized at 100 g. In contrast, the mass of tilapia fillets subjected to traditional high-temperature steaming was reduced to 88.46 ± 1.05 g. This value can be approximately converted into the actual mass of the fillets on the basis of the mass of the sample and the percentages of the respective nutrients. The outcomes are visually presented in Figure 2B. In comparison with the *sous-vide* treatment group, the moisture content of the samples cooked traditionally at high temperatures decreased by 16.62%, the protein content decreased by 17.80%, the fat content decreased by 38.41%, and the ash content decreased by 7.26%. The low heating temperature in the *sous-vide* treatment of the tilapia fillets generated less heat, and the heat generated during the cooking process led to the denaturation of myosin and actin, which caused structural changes in the myofibrillar proteins. The myoplasmic proteins in the muscle fibers migrated outward, which resulted in loss of water in the meat tissue [18]. The temperature and time of cooking mainly determine the degree of oxidation of proteins, and the oxidation products are correlated with the loss of water. A decrease in the extent of oxidation of proteins may occur because the process of cooking leads to a low loss of moisture [19]. The protein content of the control group was significantly lower than that of the *sous-vide*-treated tilapia fillets because of the degradation of proteins during the heating process, and the degradation products dissolved in the moisture present in the food and were correlated with the moisture loss. Furthermore, the samples processed by *sous-vide* treatment contained slightly more ash than the samples cooked at high temperatures. This may have been due to the transfer of inorganic compounds such as calcium, phosphorus, and potassium from the muscle to the exudate during the *sous-vide* treatment process [5]. To sum up, the *sous-vide* cooking method has been found to be successful in retaining moisture, minimizing protein denaturation and oxidation, and reducing nutrient loss in fish [14]. These results are consistent with the research conducted by Zhang et al. [20] on *sous-vide*-steamed duck meat.

### 2.3. Texture Profile Analysis of Tilapia Fillets Subjected to Different Cooking Methods

During the processing of meat, the texture of the meat can be negatively affected by heating, which results in decreases in hardness and chewiness and thus ultimately reduces the eating quality for consumers. In this study, texture profile analysis (TPA) was conducted on tilapia fillets that underwent different cooking methods. The results, which are depicted in Figure 2C, show significant differences in the hardness of tilapia fillets between *sous-vide* cooking and conventional steaming at high temperatures. Specifically, increasing the steaming temperature led to a sharp decrease in hardness and a slight decrease in springiness in tilapia fillets, and the changes in shear force when beef was cooked at 55 °C and the temperature was then increased to 65 °C showed similar results [21]. These changes in textural or sensory properties can be attributed to changes in muscle structure [22], and changes in myofibrillar proteins and connective tissue due to heating temperatures can lead to changes in the tenderness of meat during cooking [21]. The reason for the higher hardness and springiness of *sous-vide*-processed tilapia fillets could be that the heating process caused the coagulation and contraction of proteins and the thermal denaturation of the proteins led to the dehydration of muscle fibers, which made the meat harder [15]. In addition, when fish meat is subjected to heat during cooking, the proteins in the muscles are broken down. Proteases that are activated by heat, which are found in higher levels in traditionally cooked fish, bind strongly to the muscle proteins and cause the hydrolysis of myofibrillar proteins and collagen. This leads to rapid deterioration of the meat and negatively affects its gelation properties. Unlike beef, pork, and chicken, the texture of fish primarily depends on the state of myofibrillar proteins rather than collagen. This is because fish have a relatively low content of collagen-rich connective tissue [23]. Therefore, when tilapia is treated by *sous-vide* cooking, the denaturation of myofibrillar proteins is reduced, which results in the retention of hardness and springiness, as observed in the microstructure. However, the hydrophobic and hydrogen bonds that maintain the internal structure of the muscle tissue are disrupted, which causes a loss of juices and loosening of myofibrils and thus leads to a decrease in hardness. This is consistent with the changes in the secondary structure of proteins. Chewiness, which is determined by hardness, cohesion, and springiness, is improved when fish have higher hardness [24]. Therefore, *sous-vide*-treated tilapia had a better texture, as confirmed by organoleptic evaluation. Whereas cohesion is negatively correlated with hardness, chewiness, springiness, and resilience, there was no significant difference in cohesion between the cooking methods, which was consistent with the findings for duck meat [20]. *Sous-vide*-treated tilapia fillets exhibited greater springiness, hardness, and resilience, which resulted in a more favorable texture and eating experience.

### 2.4. Changes in Lipid Oxidation (TBA Value) in Tilapia Fillets Subjected to Different Cooking Methods

**Figure 3:**
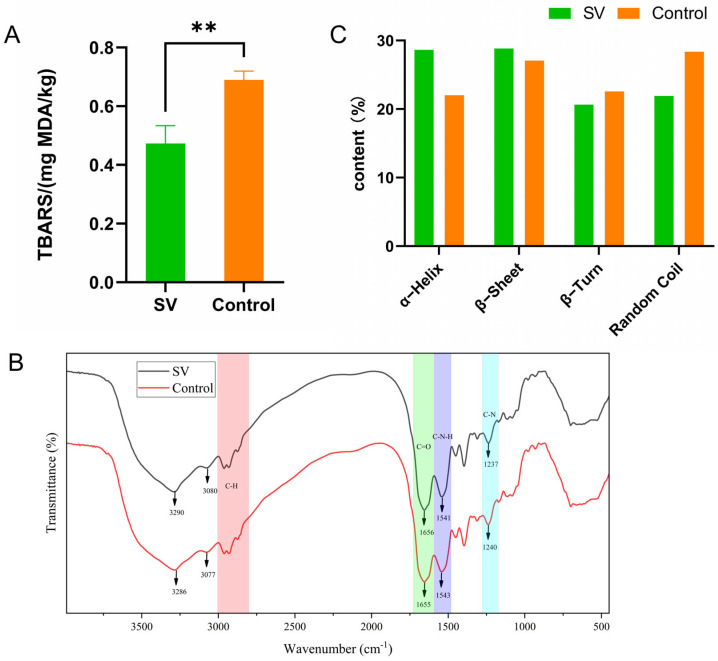
TBARS values (**A**), FTIR spectra (**B**), content of protein secondary structure (**C**) of tilapia fillets under different steaming methods. Levels of significance are shown as follows: ** 0.001 < *p* ≤ 0.01.

The TBA value represents the concentration of MDA in muscle tissue and serves as a reliable indicator of lipid oxidation [25]. The rate of lipid oxidation is influenced by the temperature during heating [26], and this process can lead to the formation of potentially harmful substances and thereby diminish the nutritional quality of the product [17]. Figure 3A displays the TBA values of tilapia fillets subjected to the different thermal processing methods and reveals that higher heating temperatures correspond to higher TBA values. Notably, the TBA value of the fillets processed using the *sous-vide* method (0.47 ± 0.06 mg MDA/kg) was significantly lower (*p* < 0.05) in comparison with the control group (0.69 ± 0.03 mg MDA/kg). This finding is consistent with the observations made regarding lipid oxidation in *sous-vide*-cooked lamb loins [26], which demonstrated that the formation of MDA is influenced by the temperature at which the meat is heated. The effect of temperature on lipid oxidation in meat can be attributed to several factors. Firstly, heating promotes the disruption of cell membranes, which facilitates interactions between polyunsaturated fatty acids and prooxidants. In addition, it induces protein denaturation and thus leads to the inactivation of antioxidant enzymes or the release of prooxidants from myoglobin, which generate further prooxidants [27]. Moreover, the formation of adducts with proteins during solubilization or the cooking process may contribute to a reduction in the MDA content [22]. The lower heating temperature employed in the *sous-vide* treatment of tilapia fillets theoretically resulted in less damage to muscle cell membranes and protein denaturation and thereby inhibited lipid oxidation. This is consistent with the changes in TBA values measured in duck [20]. The TBA values were consistent with the changes in lipid content observed in the samples, as depicted in Figure 2B. Furthermore, the presence of other volatile aldehydes, which are produced by the degradation of lipid hydroperoxides, is frequently employed as a means of assessing lipid oxidation in meat [26]. Thus, the *sous-vide* cooking method demonstrated a significant capacity to effectively inhibit the process of lipid oxidation in meat.

### 2.5. Variations in FTIR Spectra of Tilapia Fillets Subjected to Different Cooking Methods

The FTIR spectra of tilapia fillets subjected to the different cooking methods are presented in Figure 3B. The amide A band, which corresponds to the stretching vibrations of hydroxyl O–H and amino N–H bonds, exhibited a shift in frequency from 3290 cm^−1^ to 3286 cm^−1^ in the *sous-vide* treatment group, which indicated a change in hydrogen bonding during the heating process. The absorption peak at 1656 cm^−1^ corresponds to the stretching vibrations of C=O bonds in the amide I band, which play a crucial role in the formation of secondary structures in myogenic fibers [9]. The intensity of this absorption peak in the amide I band (1655 cm^−1^) was significantly reduced in the spectrum of tilapia fillets treated by conventional high-temperature cooking. In the amide II band, the FTIR spectrum of *sous-vide*-treated tilapia fillets exhibited a peak at 1541 cm^−1^. The traditional high-temperature cooking treatment caused changes in the protein structure, which resulted in a broader and lower peak at 1541 cm^−1^, which indicated the occurrence of C–N–H bending vibrations. In FTIR spectroscopy, the intensity of absorption peaks in the amide III band reflects the combined effect of irregular curling and β-structures [28]. The absorption peaks in the amide III band in the spectrum of the tilapia fillets subjected to high-temperature treatment (1239 cm^−1^) were higher than those in the spectrum of the *sous-vide* treatment group (1237 cm^−1^), which suggested that the α-helix content was higher in the group treated by conventional high-temperature steaming. This indicated a decrease in α-helical structures and an increase in random coil and β-structures in the traditional high-temperature cooking group.

FTIR spectroscopy within the amide I region (1700–1600 cm^−1^) is a technique widely used in the analysis of protein secondary structure. This region provides information on various secondary structures such as α-helix, random coil, β-turn, and β-sheet, which can be identified on the basis of distinct peaks (Figure 3B). The secondary structures of the proteins in the *sous-vide* treatment group, as shown in Figure 3C, were primarily composed of α-helix (28.63%) and β-sheet (28.83%), followed by random coil (21.92%), and β-turn (20.63%). In contrast, the secondary structures of the proteins in the samples subjected to traditional high-temperature cooking were dominated by β-sheet (27.06%) and random coil (28.37%). The α-helix structure is known for its stability and lack of cavities, which make it the most stable and prevalent secondary structure in proteins. The stability of the α-helix is primarily determined by the stability of the polypeptide chain, which in turn relies on the hydrogen bonding between the carbonyl groups and the amino groups in the polypeptide chain [28]. Under heating, proteins undergo denaturation, which results in the transformation of α-helix structures into β-sheet structures [29]. A decrease in the relative content of α-helix structures indicates a weakening of hydrogen bonding within the protein molecule and an increase in the degree of unfolding of the protein molecule [3]. In comparison with *sous-vide* treatment, traditional high-temperature steaming of tilapia fillets led to more severe denaturation of proteins, which disrupted the hydrogen bonding between carbonyl and amino groups and significantly reduced the α-helix content. The content of β-sheet structures reflects the degree of intermolecular aggregation of proteins [29]. An increase in the content of β-sheet structures promotes the formation of a well-structured protein gel and contributes to an increase in hardness in meat. The β-sheet content in the *sous-vide* treatment group was higher than that in the samples cooked at high temperatures using conventional steaming, which was consistent with the results of the textural analysis. The β-turn is a secondary structure that connects α-helix and β-sheet structures in protein molecules. The increase in β-turn content in the control group suggests that the α-helix structures gradually unraveled during traditional high-temperature cooking and may have been converted into β-turn structures. Overall, the changes in protein secondary structure indicate that *sous-vide* treatment can preserve the secondary structure and mitigate the degradation of myofibrillar proteins.

### 2.6. Sensory Evaluation of Tilapia Fillets Subjected to Different Cooking Methods

**Figure 4:**
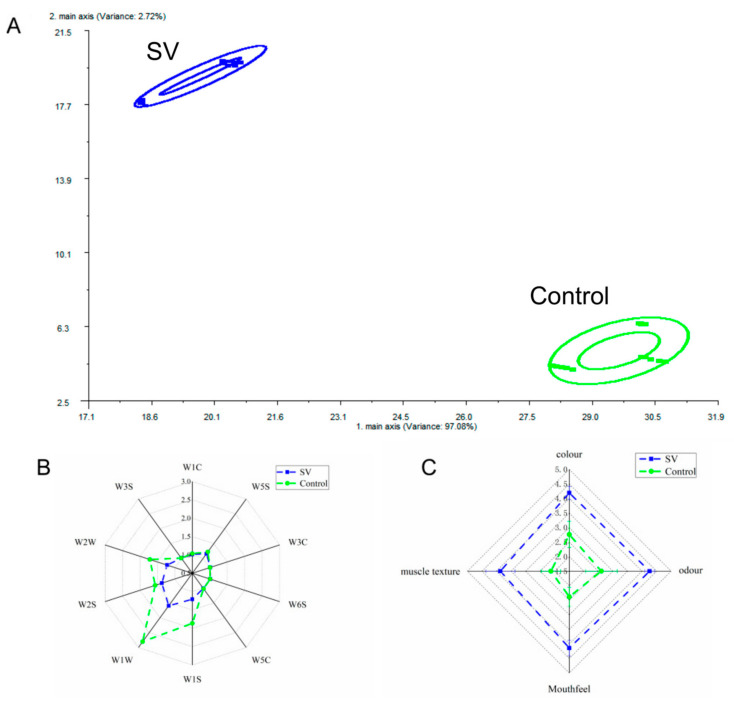
Plot of PCA scores (**A**), radar plots of e-nose analysis (**B**), sensory analysis (**C**) of tilapia fillets analyzed by e-nose for different steaming methods. The results represent the mean of six panelists on a scale ranging from 0 to 5.

The effects of the different steaming techniques on sensory properties are depicted in Figure 4C. The findings indicate that increasing the steaming temperature significantly reduced the scores for the color, odor, taste, and muscle texture of the tilapia fillet samples. The color of fish is influenced by changes in myoglobin, hemoglobin, and other color-presenting proteins. Cooking at high temperatures for extended periods causes protein denaturation, which results in the production of other derivatives. In addition, heat treatment can lead to the undesirable discoloration of seafood products as a result of the oxidation of hemoglobin and carotenoids [10]. The lower color scores observed for the tilapia fillets cooked at high temperatures were consistent with the structural changes in proteins. Conventional high-temperature steaming also led to decreases in taste and odor due to oxidation reactions between various components and oils in the food, which resulted from prolonged heating at excessively high temperatures. This indicates that high heating temperatures are not conducive to preserving the distinct fishy flavor of tilapia fillets, which is consistent with the findings of the analysis of volatile flavor compounds. The muscle texture score also significantly decreased, which was consistent with the changes observed in TPA. In contrast, the samples treated by *sous-vide* steaming exhibited significantly higher scores for color, odor, taste, and muscle texture in comparison with the samples treated by conventional high-temperature steaming (*p* < 0.05). This demonstrates that the *sous-vide* treatment method was more effective in preserving the visual quality and taste of the samples. The organoleptic quality of whiteleg shrimp tended to improve after *sous-vide* treatment at appropriate temperatures and times, which is in agreement with the findings of this study [10].

### 2.7. E-Nose Analysis of Tilapia Fillets Subjected to Different Cooking Methods

The analysis of aroma compounds using electronic nose technology is highly sensitive and capable of detecting subtle variations and differences across samples. PCA is a statistical method used to generate principal component variables that eliminates correlations between original feature variables [3]. As depicted in Figure 4A, the cumulative effect of the initial two principal components (PC1 and PC2) accounted for over 95% of the total variance. The distinct clustering of flavor compounds that resulted from the different cooking methods applied to the tilapia fillets had no overlap, which suggested that the volatile odor compounds in the fillets differed significantly under the different cooking conditions. These findings are further supported by the radar plots (Figure 4B), which reveal the contributions of array sensors W1W, W2W, W1S, and W2S to the response to the tilapia fillets, which resulted in significant responses to odors across the different samples. Specifically, sensors W1W and W2W exhibited stronger responses to sulfur-containing volatiles in tilapia fillets treated by conventional high-temperature cooking in comparison with the *sous-vide* treatment group. This suggests an enhanced odor, which can potentially be attributed to protein denaturation and oxidation and lipid oxidation resulting from prolonged heating at high temperatures. These findings are consistent with the observed changes in TBA values. Furthermore, protein degradation caused by microorganisms and endogenous enzymes can generate strong odor-producing compounds such as hydrogen sulfide, ammonia, and indole [3]. The responses of sensors W1W, W1S, W2W, and W2S in the *sous-vide* treatment group were significantly weaker than those in the conventional high-temperature treatment group (*p* < 0.05), which indicated that heating at low temperatures can reduce the generation of off-odors in fish fillets (Figure 4B). This finding also explains the superior fishy flavor observed after the *sous-vide* treatment, as confirmed by the GC–MS results. In addition, sensors W1C (aromatic compounds), W3C (ammonia, sensitive to aromatic compounds), W6S (mainly selective for hydrogen), and W3S (sensitive to long-chain alkanes) exhibited weaker signals in the samples. This suggested that aromatic compounds, ammonia, hydrides, and aliphatic compounds were poorly represented and did not differ significantly between the different cooking conditions.

### 2.8. HS–SPME–GC–MS Analysis of Tilapia Fillets Subjected to Different Cooking Methods

**Figure 5:**
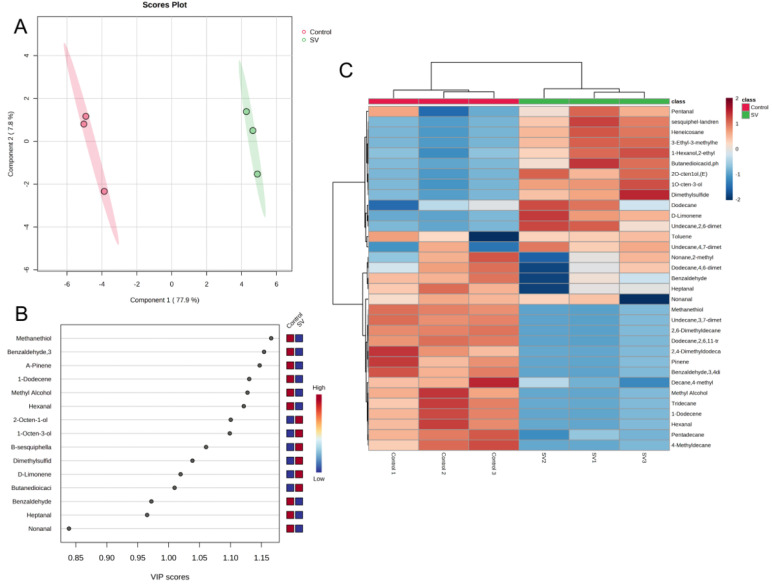
PLS–DA plot (**A**), VIP scores in PLS–DA (**B**), clustering heat map (**C**) of volatile compounds in tilapia fillets with different steaming methods.

The volatile compounds detected in the tilapia fillets processed using the different cooking methods were analyzed using GC–MS. The results are presented in Figure 5. A total of 33 volatile compounds were identified. The number of volatile compounds varied between the different cooking methods employed for the tilapia fillets. Specifically, the tilapia fillets cooked under *sous-vide* contained 21 different volatile compounds. These comprised three alcohols, two terpenes, four aldehydes, one sulfur-containing compound, ten alkanes and aromatic hydrocarbons, and one acid. On the other hand, traditional high-temperature steaming resulted in the detection of 25 volatile compounds in the tilapia fillets. These comprised two alcohols, three terpenoids, six aldehydes, one sulfur-containing compound, and 13 alkanes and aromatic hydrocarbons. Alcohols are commonly derived from fatty acids or reduced from carbonyl compounds. One specific alcohol, 1-octen-3-ol, is a product of linolenic acid degradation and contributes to mushroom flavor. It is also one of the main volatile compounds detected in fresh fish [14]. The *sous-vide* cooking method resulted in a higher relative content of 1-octen-3-ol in the tilapia fillets, which significantly contributed to the overall flavor. This suggests that *sous-vide* steaming can help to maintain the freshness of tilapia to some extent. A study conducted by Chen et al. [5] on the volatile composition of tilapia processed using different thermal processing methods further supports the importance of alcohols in flavor formation in tilapia muscle during thermal processing.

Aldehydes possess relatively low odor threshold values and are known for their green, fruity, fatty, and nutty aromas, which contribute to the overall odor of fish. These aldehydes are primarily generated via lipid oxidation processes [20]. The absence of detectable hexanal in the *sous-vide* treated samples is considered to be an indication of spoilage. Hexanal, heptanal, and nonanal, which are produced by lipid oxidation, are commonly associated with “green” odors observed in freshwater fish. These compounds are also responsible for the fishy aroma. Other volatile aldehydes that are produced via the degradation of lipid hydroperoxides are frequently employed as a means of assessing lipid oxidation in meat [26]. The rise in aldehyde levels following conventional high-temperature cooking indicates that fish fats underwent oxidation during this process. Conversely, the samples treated by the *sous-vide* method exhibited lower levels of aldehydes and ketones, as well as improved edibility, which was consistent with the observed changes in TBA values. In contrast, *sous-vide* steaming can partially mitigate the presence of fishy substances in fish. In addition, both samples contained benzaldehyde, which imparts a nutty aroma and contributes to a pleasant flavor.

The hydrocarbons present in the *sous-vide* treated tilapia fillets were primarily formed by the homogeneous cleavage of alkane radicals produced by fatty acid oxidation. The proportion of hydrocarbons in these fillets was found to be lower in comparison with the control group. It is worth noting that while hydrocarbons have higher odor thresholds and contribute less to the direct flavor of fish, they may still contribute to the overall aroma of fish [30].

Sulfur-containing compounds, which are generated by the Maillard reaction or the pyrolysis of amino acids, make a significant contribution to the aroma of fish during the heating process [5]. Methanethiol, which is a common volatile compound found in food, is characterized by its sulfur, gasoline, and garlic odors and has a detection threshold of as low as 0.02 μg/kg. Methanethiol has only been detected in tilapia fillets traditionally cooked at high temperatures, leading to the production of dimethyl sulfide [31], which has an unpleasant odor. Therefore, *sous-vide* cooking can partially inhibit the production of dimethyl sulfide and help to maintain the quality of the fish when consumed.

Heat map clustering analysis was employed to gain a deeper understanding of the distinctions between the tilapia fillets treated by the different cooking methods. The analysis utilized the relative amounts of 33 volatile compounds as variables which were normalized and compared to determine their frequency of distribution across the six samples. The results revealed that as the Euclidean distance increased, the samples could be categorized into two groups: conventional high-temperature steaming and *sous-vide* steaming. These two treatments resulted in distinct characteristic volatile compounds, as indicated by the red areas in Figure 5C. The PLS–DA score plots of volatile compounds clearly differentiated the tilapia fillets between traditional high-temperature steaming and *sous-vide* steaming on the basis of the first two principal components. In addition, the VIP scores of volatile compounds, which accounted for 80% of the cumulative contribution (Figure 5B), helped to discern the differences in volatile flavor compounds between the tilapia fillets. Among the 15 volatile compounds that made significant contributions, there were three alcohols (1-octen-3-ol, (*E*)-2-octen-1-ol, and methyl alcohol), five aldehydes (3,4-dimethylbenzaldehyde, hexanal, benzaldehyde, heptanal and nonanal), two sulfur-containing compounds (dimethyl sulfide and methanethiol), four terpenes (D-limonene, α-pinene, 1-dodecene, and β-sesquiphellandrene), and one acid (phenylsuccinic acid). These volatile compounds can be utilized to distinguish between tilapia fillets cooked using *sous-vide* steaming and conventional high-temperature steaming.

### 2.9. HS–GC–IMS Analysis of Tilapia Fillets Subjected to Different Cooking Methods

**Figure 6:**
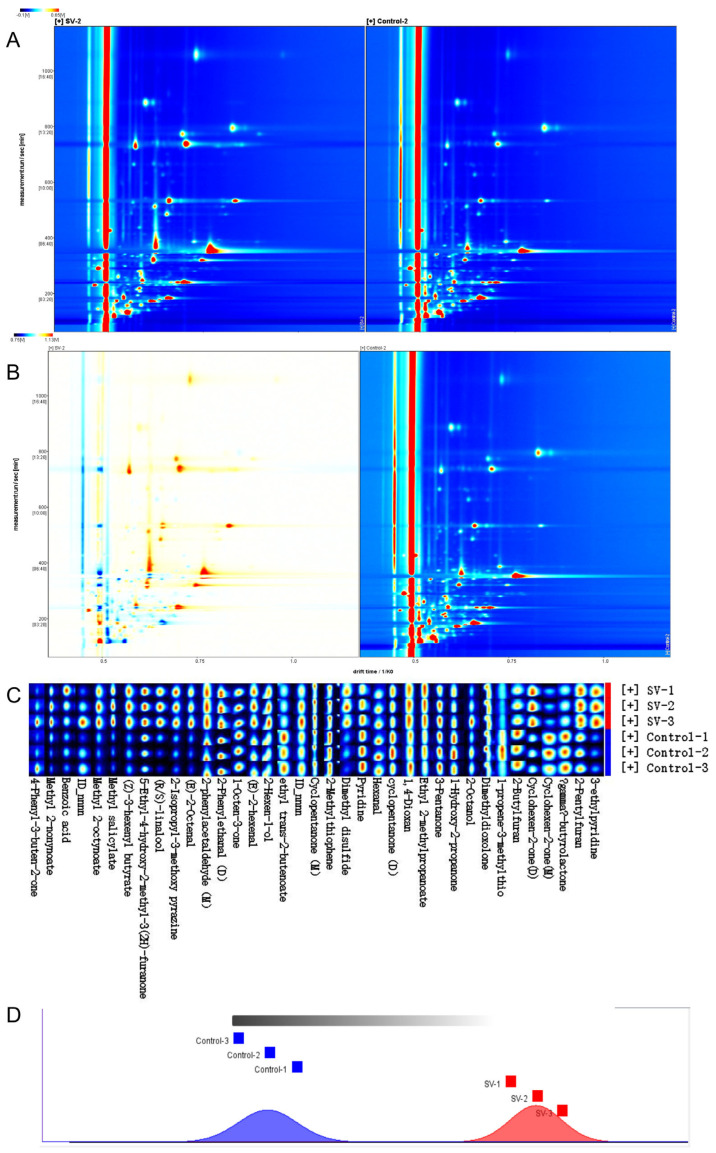
(**A**) Topographic plot of volatile fingerprints in tilapia fillets from different steaming methods, (**B**) obtained by subtracting the conventional high-temperature-treated muscle from the *sous-vide* treated muscle. The redder the area, the greater the amount of VOC. (**C**) Comparison of the fingerprints of volatile organic compounds of tilapia fillets with different cooking methods. The redder the area, the higher the amount of volatile organic compounds. Each row represents all signals selected in a sample. Each column represents signals of the same volatile organic compounds. (M) and (D) represent monomer and dimer, respectively. (**D**) Fingerprint similarity analysis of volatile organic compounds in tilapia fillets with different cooking methods.

HS–GC–IMS was employed to analyze the VOCs present in the tilapia fillets that underwent the different cooking techniques. The obtained results are depicted in Figure 6, which includes a representative topographic map (Figure 6A) and a topographic subtraction map (Figure 6B). The latter was obtained by subtracting the signals of the samples treated by conventional high-temperature steaming from those of the samples treated by the *sous-vide* method. A total of 37 regions were identified within the muscle tissue, of which each exhibited distinct retention times (*y*-axis), relative drift times (*x*-axis), and signal intensities (indicated by color intensities, with redder regions representing higher signal intensities) [32]. The VOCs were identified by comparing their corresponding drift times and retention indices with those in the NIST library. Regarding the 37 identified regions, 35 compounds were successfully identified, while two compounds remained unidentified. Furthermore, the monomeric and dimeric forms of three compounds (2-phenylacetaldehyde, cyclopentanone, and 2-cyclohexenone) were detected using HS–GC–IMS. Consequently, a total of 32 VOCs were identified in the tilapia fillets treated by the different cooking methods. These VOCs consisted of four alcohols, four aldehydes, eight ketones, seven esters, two sulfur-containing compounds, three nitrogen-containing compounds, and four other compounds (Table S2).

In order to analyze the changes in volatile compounds, fingerprints were generated using all the peaks (Figure 6C) to further examine the flavor compounds present in the tilapia fillets cooked using the different methods. Each sample was tested in triplicate to ensure accuracy. The graph displays the volatile compounds found in all samples, and each column allows a comparison of the differences in the content of the same volatile compound. Lighter colors on the graph indicate lower levels of the corresponding compounds. The FSA (Functional Similarity Analysis) plugin is a novel plugin for the HS–GC–IMS software (version VOCal-0.4.03) that facilitates the analysis of VOC fingerprints by calculating and comparing Euclidean distances (Figure 6D). The larger is the distance between samples in the figure, the more pronounced were the differences between the samples.

In the samples analyzed in this study, aldehydes were present in the highest relative content. Aldehydes are primarily formed by the oxidative breakdown of fatty acids. Their low odor threshold values suggest that aldehydes had a significant effect on the overall flavor of the fish samples. Notably, the content of aldehydes in the tilapia fillets subjected to *sous-vide* treatment was significantly higher in comparison with those treated by traditional high-temperature steaming. Phenylacetaldehyde (as both a monomer and a dimer) was found to have distinct green and apricot flavors and had the highest relative content among the aldehydes identified [31]. Olefinic aldehydes, such as (*E*)-2-octenal and (*E*)-2-hexenal, contribute to the fatty odor of fish and are primarily produced by the degradation of linoleic and linolenic acids. Hexenal, in particular, is the main component responsible for fishy odor and is predominantly found in areas of fish with high fat contents [33]. The conventional high-temperature treatment group exhibited higher levels of hexenal in comparison with the *sous-vide* group, which indicated that high-temperature heating led to a greater extent of lipid oxidation and the production of more unpleasant odors. This finding was consistent with the observed changes in TBA values. Cyclopentanone is characterized by a minty odor, while diketones have sweet, buttery, and caramel flavors. In comparison with aldehydes and alcohols, the difference in ketones between the two treatment groups was smaller, but their overall relative content was higher. This suggests that ketones contributed to reducing the fishy odor and made positive contributions to the overall flavor of the fish samples.

Esters are commonly associated with the development of fruity flavors and can be synthesized by the reaction of free fatty acids and alcohols or via transesterification reactions involving fatty acids in triglycerides and ethanol [20]. In the *sous-vide* treatment group, the levels of methyl 2-nonynoate, methyl 2-octynoate, methyl salicylate, ethyl trans-2-butenoate, and ethyl 2-methylpropanoate were higher than those in the group treated by conventional high-temperature steaming. The presence of esters in the tilapia fillets enriched the flavor compounds and improved the overall quality of the samples. In addition, 1-octen-3-ol was found in higher relative content in the *sous-vide* treated tilapia fillets than in the conventional high-temperature treatment group, which was consistent with the results obtained from GC–MS analysis. Notably, the content of 2-isopropyl-3-methoxypyrazine differed significantly between the two groups, and the *sous-vide* group exhibited significantly higher levels. This compound is known to contribute to green pea odor in tilapia fillets. Furthermore, the levels of 2-butylfuran and 2-pentylfuran were observed to be elevated in tilapia fillets treated with the *sous-vide* cooking method. These compounds are known to be significant contributors to the flavor profile of protein products, as they impart a pleasant combination of sweetness, meatiness, and caramelization. The formation of furans typically occurs through the thermal breakdown of carbohydrates, the Maillard reaction involving amino acids and reducing sugars, and the oxidation of lipids [34]. Therefore, the *sous-vide* treated tilapia fillets were enriched with flavor compounds, but the specific mechanism of the formation of volatile compounds needs further study.

## 3. Materials and Methods

### 3.1. Sample Preparation

Tilapia with an average weight of 0.85 ± 0.25 kg were purchased from Nanbin Farmers Market in Sanya, Hainan, China. The fish were transported to the laboratory and within 30 min were cut into fillets with an average weight of 150 ± 25 g. Each fillet was individually packed in a vacuum bag, which was sealed using a vacuum packaging machine (DZQ–6002SB, Ruibao Packaging Machinery Co., Ltd., Guangzhou, China). Then, the fillets were divided into two groups of three pieces each and placed in a transparent constant-temperature tank (Bilon–HT–HI, Bilon Instrument Manufacturing Co., Ltd., Shanghai, China). Treatment was carried out at 65 °C for 45 min for the *sous-vide* group and at 100 °C for 45 min for the control group. The samples that were heated were removed and rapidly cooled to a temperature below 10 °C using ice water. They were then placed in a refrigerator at 4.0 ± 0.5 °C for future analysis.

### 3.2. Proximate Composition Analysis

The crude protein and fat contents were determined by the micro-Kjeldahl and Soxhlet extraction methods, respectively, according to the AOAC standards [35]. Moisture was determined using the AOAC air oven method [35] by weighing out a 2.00 ± 0.01 g sample and drying it in an oven at 100 ± 2 °C for more than 18 h. Ash was determined using the basic AOAC method [35] by heating a 3.00 ± 0.02 g sample in an oven at 550 °C for 10 ± 2 h.

### 3.3. Microstructural Analysis by Optical Microscopy

Tilapia muscle was cut into small cubes with dimensions of 5 × 5 × 5 mm and then fixed in a solution containing 4% paraformaldehyde. After fixation, the samples were dehydrated using a mixture of phosphate-buffered saline and methanol with increasing concentrations. Each dehydration step lasted 30 min. Following dehydration, the fish samples were sequentially immersed in anhydrous ethanol, a mixture of anhydrous ethanol and xylene in a 1:1 ratio, and pure xylene for 15 min each time. After dehydration, the fish samples were immersed in pure paraffin wax twice for 1 h each time. Once the samples were transparent, they were placed in a plastic box and embedded in melted paraffin wax [5]. After the paraffin-embedded samples had cooled, cross-sections with a thickness of 40 μm were cut using a rotary slicer (RM5, Leica, Shanghai, China). These sections were then spread out on clean slides and dried overnight on a roaster at 45 °C. To prepare the sections for observation, they were deparaffinized using xylene and a series of ethanol solutions with increasing concentrations. The sections were then rinsed with distilled water and stained with hematoxylin and eosin (H&E). Finally, the sections were observed using an optical microscope (Leica, Wetzlar, Germany).

### 3.4. Scanning Electron Microscopy Measurements

Tilapia fillets were cut into samples with dimensions of 2 × 2 × 2 cm. These samples were then fixed in a 2.5% glutaraldehyde solution at 4 °C for 12 h [36]. After the initial fixation, the samples were removed and trimmed to dimensions of approximately 0.5 × 0.5 × 0.5 cm. They were then fixed for a second time and dehydrated using ethanol solutions of various concentrations. Following this, the samples were lyophilized in a refrigerator overnight at −80 °C and subsequently freeze-dried. The freeze-dried samples were coated with gold and observed and photographed using a scanning electron microscope (Quanta FEG250, FEI, Hillsborough, OR, USA).

### 3.5. Measurement of Texture Profile Analysis

A sample was cut into pieces measuring 5 × 5 × 5 cm to analyze their TPA (TA. XT. Plus C, Stable Micro Systems, Surrey, UK). A cylindrical probe with a diameter of 2 mm was used to measure the hardness (g), springiness (mm), cohesiveness (mm), chewiness, and resilience (mm) of the sample. The measurement parameters were as follows: the test speed was 60 mm/min, the trigger force was 5.0 g, the compression rate was 40%, the probe return speed after the measurement was 100 mm/min, and the probe height was 60 mm. Each sample was tested five times.

### 3.6. Measurement of Thiobarbituric Acid Value

A 5.00 ± 0.01 g sample of tilapia was accurately weighed, shaken with a trichloroacetic acid (TCA) solution at 50 °C for 30 min, and then filtered through double-layer quantitative filter paper. A 5 mL sample of the filtrate was mixed with the same volume of thiobarbituric acid (TBA) (0.02 mol/L) and shaken thoroughly, and then the mixture was heated at 90 °C in a water bath for 30 min and cooled to room temperature. The absorbance at 532 nm was measured, and the TCA blank was used as the control. The concentration of TBA (mg/kg) was calculated from the malondialdehyde (MDA) standard curve.

### 3.7. Fourier Transform Infrared Spectroscopy and Secondary Structure Analysis

A sample and water were homogenized at 12,000 rpm in a 1:4 ratio and subsequently lyophilized. A quantity of 2 ± 0.5 mg of the lyophilized sample was then placed in an agate mortar and mixed with 15–25 mg dried KBr crystals. The mixture was ground into a powder and pressed into a transparent tablet using a tablet press [37]. Then, the Fourier transform infrared (FTIR) spectrum of the sample was recorded at a resolution of 4 cm^−1^ over the wavelength range of 4000–400 cm^−1^ (Nicolet iS 5, Thermo Fisher, Waltham, MA, USA), and the number of scans was 64. The obtained data were analyzed using PeakFit 4.12 software (Systat Software, Inc., San Jose, CA, USA) on the basis of a Gaussian distribution.

### 3.8. E-Nose Analysis

The volatile compounds present in samples were analyzed using an electronic nose system (PEN3.0, AIRSENSE, Schwerin, Mecklenburg, Germany) that was equipped with ten sensors. A 1.00 ± 0.05 g portion of a sample was placed in a 10 mL glass injection vial and allowed to reach equilibrium at room temperature for 30 min. The sensor cleaning process lasted 60 s, while the auto-zeroing process took 10 s. Sample preparation was completed within 5 s, and the detection process lasted 120 s. Data analysis was conducted using the stable response curve observed between 105 s and 108 s.

### 3.9. Sensory Evaluation

A sensory evaluation panel consisting of six individuals with expertise in the field of food (age range 22–25 years, gender ratio 1:1) was assembled. Tilapia fillet samples were presented on white plates at ambient temperature and were randomly allocated to the panel members. The panel members assessed the color, odor, taste, and muscle texture of the tilapia fillets, as outlined in Table S1.

### 3.10. HS–SPME–GC–MS Analysis

A sample was divided into pieces with a length of approximately 3 mm and a weight of 5.00 g. These pieces were placed in a 20 mL headspace vial with 5 mL of an NaCl solution with a concentration of 0.18 g/mL and 5 μL of a methanol solution containing the internal standard 2,4,6-trimethylpyridine at a concentration of 10 mg/g [38]. The vial was immediately covered and inserted into a solid-phase extraction headspace injection device (Zhenzheng ZZ–SPME–Bath, Zhenzheng Analytical Instrument Co., Ltd., Qingdao, China) set at 50 °C without stirring. The extraction handle was inserted into the device, and the carboxen/polydimethylsiloxane/divinylbenzene SPME head was extended to a distance of approximately 10 mm from the sample. The sample was exposed to high temperatures for 50 min at 50 °C. After the completion of adsorption, the fiber tip was removed from the sample and immediately inserted into the GC–MS injection port. The sample was held at 250 °C for 5 min and thermally desorbed for GC–MS detection [5]. Each sample was tested three times.

GC was conducted using an HP–5ms column (30 m × 0.25 mm × 0.25 μm; Agilent Technologies, Inc., Santa Clara, CA, USA). The column temperature was initially set at 40 °C and held for 3 min. Subsequently, the temperature was increased to 60 °C at 5 °C/min and held for 3 min. It was then further increased to 200 °C at 6 °C/min and held for 3 min. Finally, the temperature was raised to 250 °C at 8 °C/min. The helium flow rate was 1 mL/min. MS was performed in electron impact mode with a detector voltage of 1.2 kV and a mass-to-charge ratio ranging from 45 to 550 *m*/*z*. MS spectra were obtained at an ion source temperature of 230 °C, a quadrupole temperature of 150 °C, and a transmission line temperature of 280 °C.

The volatile compounds were identified based on their mass spectra and retention indices. The retention indices were calculated with reference to Zhou et al. [39] using the C6–C24 n-alkane series. The volatile compounds were determined using MassHunter Workstation10.0 software unknowns’ analysis and searched in the NIST 20.L library according to the minimum matching factor of 85. 

The relative content of each volatile chemical was determined by calculating the ratio of its peak area to the peak area of the internal standard (2,4,6-trimethylpyridine). The relative contents (μg/kg) of each volatile compound in tilapia fillets cooked using the different methods was calculated as follows [17]:

Compound relative content = (internal standard concentration/peak area of internal standard) × peak area of compound(1)

### 3.11. HS–GC–IMS Analysis

A 3.00 g sample of vacuum-cooked tilapia fillets was weighed out, put into a 20 mL headspace bottle, and subjected to HS–GC–IMS (Agilent 490 gas chromatograph, Agilent Technologies, Palo Alto, CA, USA; FlavourSpec, G.A.S., Dortmund, Germany) for the analysis of volatile organic compounds (VOCs) released from fragments of tilapia muscle. The operating conditions of the HS–GC–IMS device were as follows: analysis time, 25 min; column type, MXT–5; column size, 15 m, 0.53 mm i.d.; column temperature, 60 °C; carrier gas/drift gas, N_2_; and IMS temperature, 45 °C. The operating conditions of the automatic headspace injection unit were as follows: injection volume, 500 μL; incubation time, 15 min; incubation temperature, 60 °C; injection needle temperature, 85 °C; and oscillation rate, 500 rpm. The operating conditions of the GC device were as follows: drift gas velocity, 150 mL/min; and the program increased the carrier gas velocity by 2 mL/min (0 min), 10 mL/min (10 min), 100 mL/min (20 min), and 150 mL/min (25 min). Each sample was tested three times.

Data analysis was carried out using commercial VOCal-0.4.03 software from G.A.S. with four plugins (Reporter, gallery 1.2.8, Dynamic PCA, and FSA). The retention indices (RI) of the volatiles in the tilapia fillet samples were corrected by using the curves obtained under the same analytical conditions with ortho ketones (C4–C9) as the external standard as the reference. The RI and drift time (Rt) of the compounds in the GC-IMS Library were compared to characterize the volatiles. The Reporter plugin was used to display topographic maps and compare spectral differences to obtain topographic subtraction maps. In the topographic maps, each region (point, peak) represented a VOC, which was characterized by its retention time (on the *y*-axis), relative drift time (on the *x*-axis), and signal strength. The gallery 1.2.8 plugin was used to compare fingerprint spectra and analyze differences in volatile compounds. The built-in NIST2014 and IMS databases were used for qualitative analysis of volatile compounds.

### 3.12. Statistical Analysis

The results were reported as the mean ± standard deviation. The data obtained were subjected to statistical analysis using one-way analysis of variance with SPSS Statistics 22.0 software. Statistical analyses for the differences were performed using GraphPad Prism 9.5.1 (GraphPad Software, CA, USA). Statistical significance analysis was performed by *t*-tests (*p* < 0.05) in GraphPad Prism. The HS–SPME–GC–MS data were analyzed using MetaboAnalyst 5.0 (https://www.metaboanalyst.ca/, accessed on 1 August 2023.), specifically employing partial least squares discriminant analysis (PLS–DA), variable importance in projection (VIP) scores, and heat map clustering. The HS–GC–IMS data were analyzed using principal component analysis (PCA) and fingerprint similarity analysis with VOCal-0.04.03 software.

## 4. Conclusions

The findings of this study demonstrate that the different steaming methods had a significant effect on the physicochemical, textural, and flavor characteristics of the tilapia fillets. In comparison with the traditional high-temperature steaming method, the use of the *sous-vide* technique resulted in tilapia fillets with a more uniform and compact microstructure. This technique also minimized damage to the tissue structure of the fish flesh and resulted in fillets with greater springiness (0.58 ± 0.02 mm), hardness (123.19 ± 5.83 g), and resilience (0.14 ± 0.02 mm). In addition, *sous-vide* cooking helped to retain more nutrients in the fillets, which led to an enhanced eating experience. Furthermore, the *sous-vide* method reduced the extent of lipid oxidation in the tilapia fillets and increased the α-helix content (28.63%) in comparison with traditional high-temperature steaming. These findings suggest that sous-vide treatment effectively inhibits protein oxidation and preserves the desired flavor characteristics. The sensory evaluation and analysis of volatile compounds indicated that steaming tilapia fillets using the *sous-vide* method maintained the freshness of the ingredients while reducing fishy taste and unpleasant odors. In conclusion, the application of the sous-vide steaming technique to tilapia fillets maximizes taste and textural properties, minimizes nutrient loss, preserves the original flavor, and improves the overall eating quality. However, further investigation is necessary in order to conduct a more comprehensive study, particularly because of the lack of knowledge regarding the intricate molecular mechanisms underlying the alterations in volatile compounds and the refrigerated shelf life of tilapia fillets treated by the sous-vide method.

## Data Availability

Data will be made available on request.

## Supplementary Materials

The following supporting information can be downloaded at: https://www.mdpi.com/article/10.3390/molecules28248075/s1, Table S1: Description of sensory analysis and assessment scores; Table S2: VOCs detected in tilapia fillets of different cooking methods by HS-GC-IMS.
