# Supplementary material for: Effects of Sous-Vide on Quality, Structure and Flavor Characteristics of Tilapia Fillets
Source: Molecules. 2023 Dec 13;28(24):8075. doi: 10.3390/molecules28248075 (PMC10745649; doi:10.3390/molecules28248075)
Supplement: Supplementary file 1 [file molecules-28-08075-s001.zip › molecules-2712488-supplementary.pdf]

Supplementary Material

Table S1 Description of sensory analysis and assessment scores

score	Colour and lustre	odor	Taste	Muscle texture
5	The muscle cuts exhibit a glossy appearance with a normal coloration	With tilapia unique aroma, no fishy odor	The meat is tender, chewable and palatable	High hardness, elastic, finger pressure after the depression disappeared immediately
4	The coloration appears to be within the expected range, while the muscle cuts exhibit a glossy appearance.	The characteristic aroma is light, slightly fishy	The meat is tender, chewable and palatability is good	High hardness, slightly elastic, finger pressure after the depression disappeared faster
3	Colour a little dull, muscle cuts slightly shiny	Slightly fishy	The meat is loose, chewability is good, palatability is general	A little hardness, elastic, finger pressure after the depression disappear slightly slow

2	Dull in colour, muscle cuts almost lustreless	No aroma, has a heavy fishy smell	The meat is looser, less chewable and less palatable	The hardness is low, almost inelastic, and the depression disappears slowly after finger pressure
1	The color is dull, the muscle section is dull	Has a strong fishy or ammonia odor	The meat is loose, with low mastication and poor palatability	No hardness, no elasticity, finger pressure after the depression almost does not disappear

Table S2 VOCs detected in tilapia fillets of different cooking methods by HS-GC-IMS.

Volatile compounds	CAS	Formula	Molecular weight	RI	Rt [sec]	Dt [a.u.]	Peak Volume (a.u.)	
							Sous-vide	Control
**Alcohols**								
2-Octanol	C123966	C8H18O	130.2	1027.5	387.388	1.46986	1146.78±16.58[a]	996.44±70.07[b]
2-Hexen-1-ol	C2305217	C6H12O	100.2	866.3	219.691	1.18224	2338.23±634.94[a]	1313.76±195.17[b]
(R/S)-linalool	C78706	C10H18O	154.3	1105.8	535.135	1.69025	3083.36±907.25[a]	3095.06±848.85[a]

1-Octen-3-ol	C3391864	C8H16O	128.2	990.6	333.821	1.1706	620.45±33.14[a]	573.25±6.68[b]
**Aldehydes**								
2-phenylacetaldehyde（M）	C122781	C8H8O	120.2	1017.3	371.503	1.26663	2341.83±138.03[a]	618.82±273.02[b]
2-phenylacetaldehyde（D）	C122781	C8H8O	120.2	1007.7	357.132	1.55663	1312.43±56.63[a]	508.57±49.72[b]
(E)-2-Octenal	C2548870	C8H14O	126.2	1084	489.075	1.32519	3757.31±34.86[a]	2267.79±441.19[b]
(E)-2-hexenal	C6728263	C6H10O	98.1	906.9	247.977	1.1957	1676.43±25.52[a]	1531.88±33.53[a]
Hexanal	C66251	C6H12O	100.2	789.7	179.214	1.28669	750.23±12.94[a]	1150.36±64.81[b]
**Ketones**								
4-Phenyl-3-buten-2-one	C122576	C10H10O	146.2	1331.3	1356.422	1.82127	3083.36±907.25[a]	3095.06±848.85[a]
Cyclopentanone（M）	C120923	C5H8O	84.1	803	185.664	1.33282	1317.91±68.45[a]	1091.30±59.73[a]
Cyclopentanone（D）	C120923	C5H8O	84.1	803.2	185.753	1.09584	6093.79±167.96[a]	2901.20±96.15[b]
2-Butanone, 3-hydroxy-	C513860	C4H8O2	88.1	724.4	147.871	1.0382	475.11±10.56[a]	723.44±77.23[b]
3-Pentanone	C96220	C5H10O	86.1	687.5	132.556	1.11364	450.49±57.34[a]	494.67±28.41[a]
1-Hydroxy-2-propanone	C116096	C3H6O2	74.1	653.3	119.766	1.04505	5926.76±705.35[a]	7216.39±1199.21[b]
5-Ethyl-4-hydroxy-2-methyl-3(2H)-furanone	C27538096	C7H10O3	142.2	1106.2	536.091	1.33558	368.48±1.88[a]	482.60±33.56[a]
Dimethyldioxolone	C37830903	C5H6O3	114.1	942.7	281.586	1.18329	1676.43±25.52[a]	1531.88±33.53[a]

Cyclohexen-2-one(D)	C930687	C6H8O	96.1	900.0	241.918	1.41822	5774.54±197.27[a]	2652.78±86.66[b]
Cyclohexen-2-one(M)	C930687	C6H8O	96.1	890.2	234.059	1.11859	111.74±5.46[a]	278.87±29.81[a]
**Esters**								
Methyl 2-nonynoate	C111808	C10H16O2	168.2	1271.1	1057.899	1.48003	3278.29±546.75[a]	1640.06±458.32[b]
Methyl 2-octynoate	C111126	C9H14O2	154.2	1195.5	774.534	1.40909	1867.83±109.19[a]	759.03±105.82[b]
Methyl salicylate	C119368	C8H8O3	152.1	1181.8	732.223	1.16124	3415.00±227.22[a]	1349.97±89.05[b]
ethyl trans-2-butenoate	C623701	C6H10O2	114.1	847.5	208.98	1.18395	1479.19±17.18[a]	1091.91±3.72[b]
(Z)-3-hexenyl butyrate	C16491364	C10H18O2	170.3	1184.4	739.954	1.42843	1197.09±160.59[a]	1799.47±370.09[b]
ethyl 2-methylpropanoate	C97621	C6H12O2	116.2	750.9	159.97	1.1911	740.51±30.55[a]	579.99±52.29[a]
gamma-butyrolactone	C96480	C4H6O2	86.1	922.8	262.321	1.06507	618.09±15.47[a]	1047.02±66.55[b]
**S-containing compounds**								
Dimethyl disulfide	C624920	C2H6S2	94.2	768.8	168.717	1.13454	740.51±30.55[a]	579.98±52.29[b]
2-Methylthiophene	C554143	C5H6S	98.2	785.9	177.411	1.06102	1560.78±31.99[a]	1835.96±75.91[b]
**N-containing compounds**								
2-Isopropyl-3-methoxy pyrazine	C25773404	C8H12N2O	152.2	1095.9	513.767	1.26197	4902.16±138.13[a]	1940.32±153.17[b]
Pyridine	C110861	C5H5N	79.1	780.2	174.505	1.24383	3432.7±286.01[a]	3609.7±256.05[a]
3-ethylpyridine	C536787	C7H9N	107.2	978.9	320.296	1.51183	2057.46±132.35[a]	486.69±22.70[b]

**Others**								
1,4-Dioxan	C123911	$C_4H_8O_2$	88.1	747.7	158.462	1.11359	5586.19±583.77[a]	7455.20±814.74[b]
Benzoic acid	C65850	$C_7H_6O_2$	122.1	1229.1	889.933	1.20995	2338.23±634.95[a]	1313.76±195.17[b]
2-Butylfuran	C4466244	$C_8H_{12}O$	124.2	893.2	236.153	1.19585	368.48±1.88[a]	482.60±33.56[a]
2-Pentylfuran	C3777693	$C_9H_{14}O$	138.2	981.3	322.983	1.25623	3410.78±82.50[a]	1807.21±36.82[b]
**Unidentified**								
ID_nnnn	unidentified	*	0	1202	795.689	1.67553	2573.47±1505.21[a]	2046.52±961.95[b]
ID_nnnn	unidentified	*	0	824.2	196.417	1.17146	252.98±32.49[a]	226.17±27.23[a]

Note: Different superscript letters within the same row indicate significant differences among samples ($p < 0.05$)
